# Author Correction: Spatial organization of the mouse retina at single cell resolution by MERFISH

**DOI:** 10.1038/s41467-023-41825-2

**Published:** 2023-09-28

**Authors:** Jongsu Choi, Jin Li, Salma Ferdous, Qingnan Liang, Jeffrey R. Moffitt, Rui Chen

**Affiliations:** 1 Department of Biochemistry and Molecular Biology, Baylor College of Medicine, Houston, TX 77030 USA; 2 Department of Molecular and Human Genetics, Baylor College of Medicine, Houston, TX 77030 USA; 3 Program in Cellular and Molecular Medicine, Boston Children’s Hospital; Department of Microbiology, Harvard Medical School, Boston, MA 02115 USA

**Keywords:** Retina, Genetics of the nervous system

Correction to: *Nature Communications* 10.1038/s41467-023-40674-3, published online 15 August 2023

In this article, Jongsu Choi and Jin Li should have been denoted as equally contributing authors. The original article has been corrected.

