# Supplementary material for: Spatial organization of the mouse retina at single cell resolution by MERFISH
Source: Nat Commun. 2023 Aug 15;14:4929. doi: 10.1038/s41467-023-40674-3 (PMC10427710; doi:10.1038/s41467-023-40674-3)
Supplement: Supplementary file 1 — Supplementary Information [file 41467_2023_40674_MOESM1_ESM.pdf]

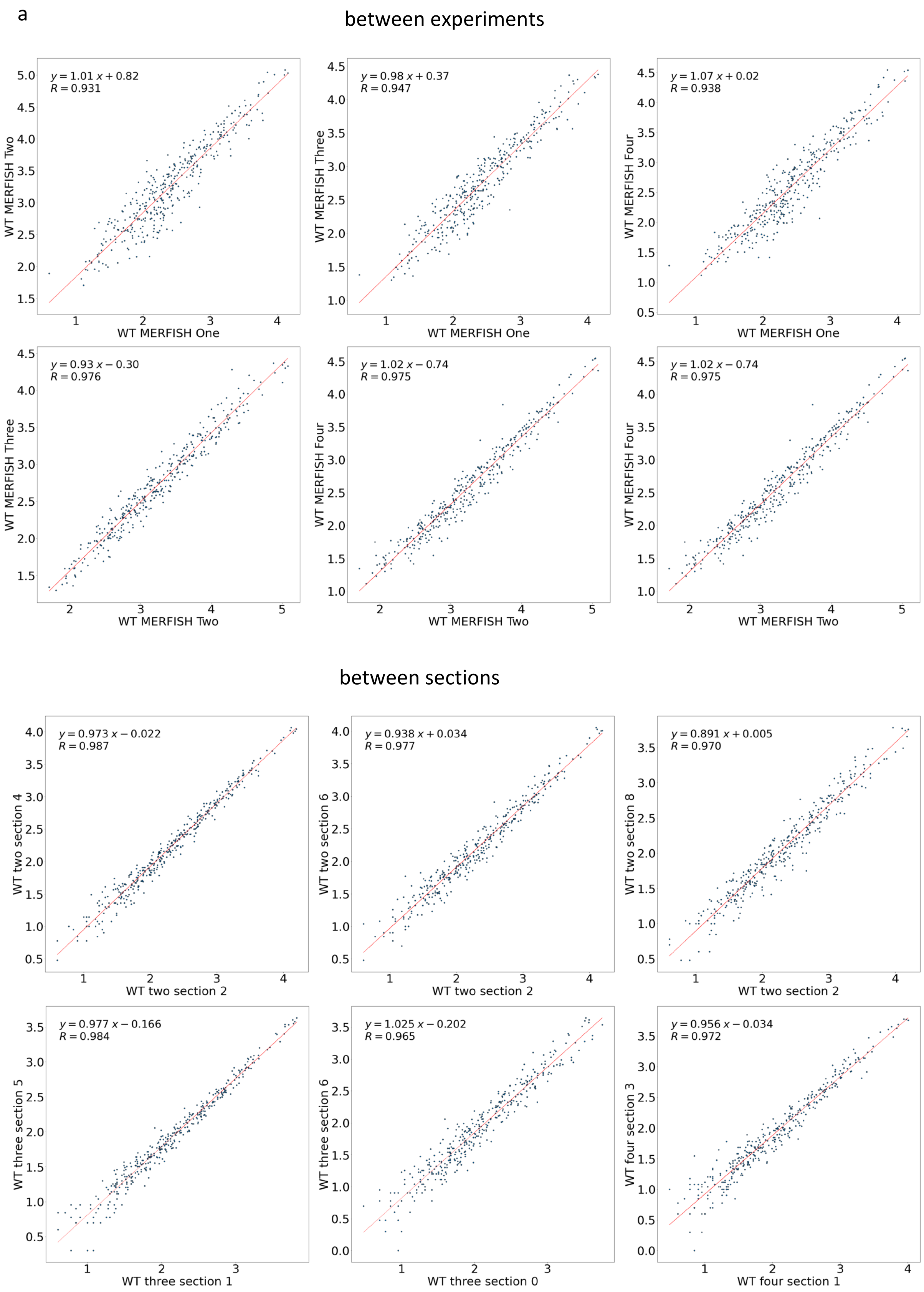
a
between experiments
y = 1.01 x + 0.82
R = 0.931
WT MERFISH Two
WT MERFISH One
y = 0.98 x + 0.37
R = 0.947
WT MERFISH Three
WT MERFISH One
y = 1.07 x + 0.02
R = 0.938
WT MERFISH Four
WT MERFISH One
y = 0.93 x − 0.30
R = 0.976
WT MERFISH Three
WT MERFISH Two
y = 1.02 x − 0.74
R = 0.975
WT MERFISH Four
WT MERFISH Two
y = 1.02 x − 0.74
R = 0.975
WT MERFISH Four
WT MERFISH Two
between sections
y = 0.973 x − 0.022
R = 0.987
WT two section 4
WT two section 2
y = 0.938 x + 0.034
R = 0.977
WT two section 6
WT two section 2
y = 0.891 x + 0.005
R = 0.970
WT two section 8
WT two section 2
y = 0.977 x − 0.166
R = 0.984
WT three section 5
WT three section 1
y = 1.025 x − 0.202
R = 0.965
WT three section 6
WT three section 0
y = 0.956 x − 0.034
R = 0.972
WT four section 3
WT four section 1

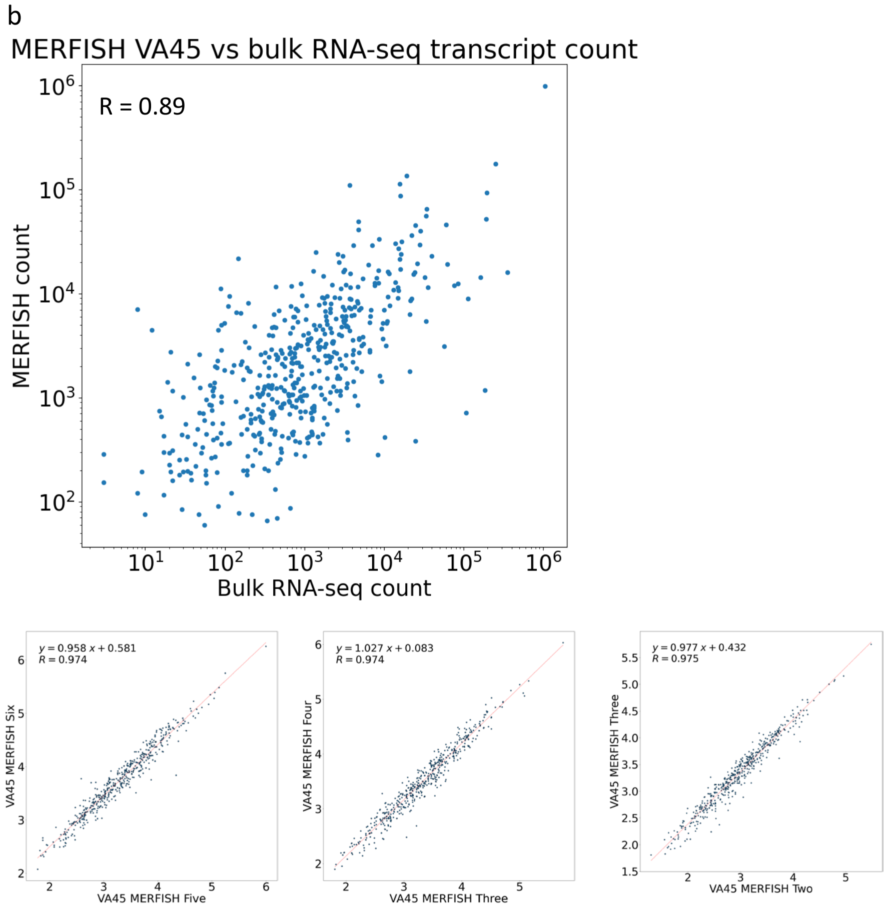

Supplementary Figure 1. Reproducibility of MERFISH data. a, Pearson correlation coefficients between four MERFISH experiment (368 gene panel) replicates and between retina tissue sections within the same experiment. High reproducibility can be observed between tissue sections and experimental replicates. **b,** Transcript count comparison between MERFISH (500 gene panel) and bulk RNA-seq and Pearson correlation coefficient between MERFISH replicates (500 gene panel). A high correlation shows MERFISH transcription detection level is comparable to RNA-seq. High reproducibility was observed between experimental replicates using the 500 gene panel.

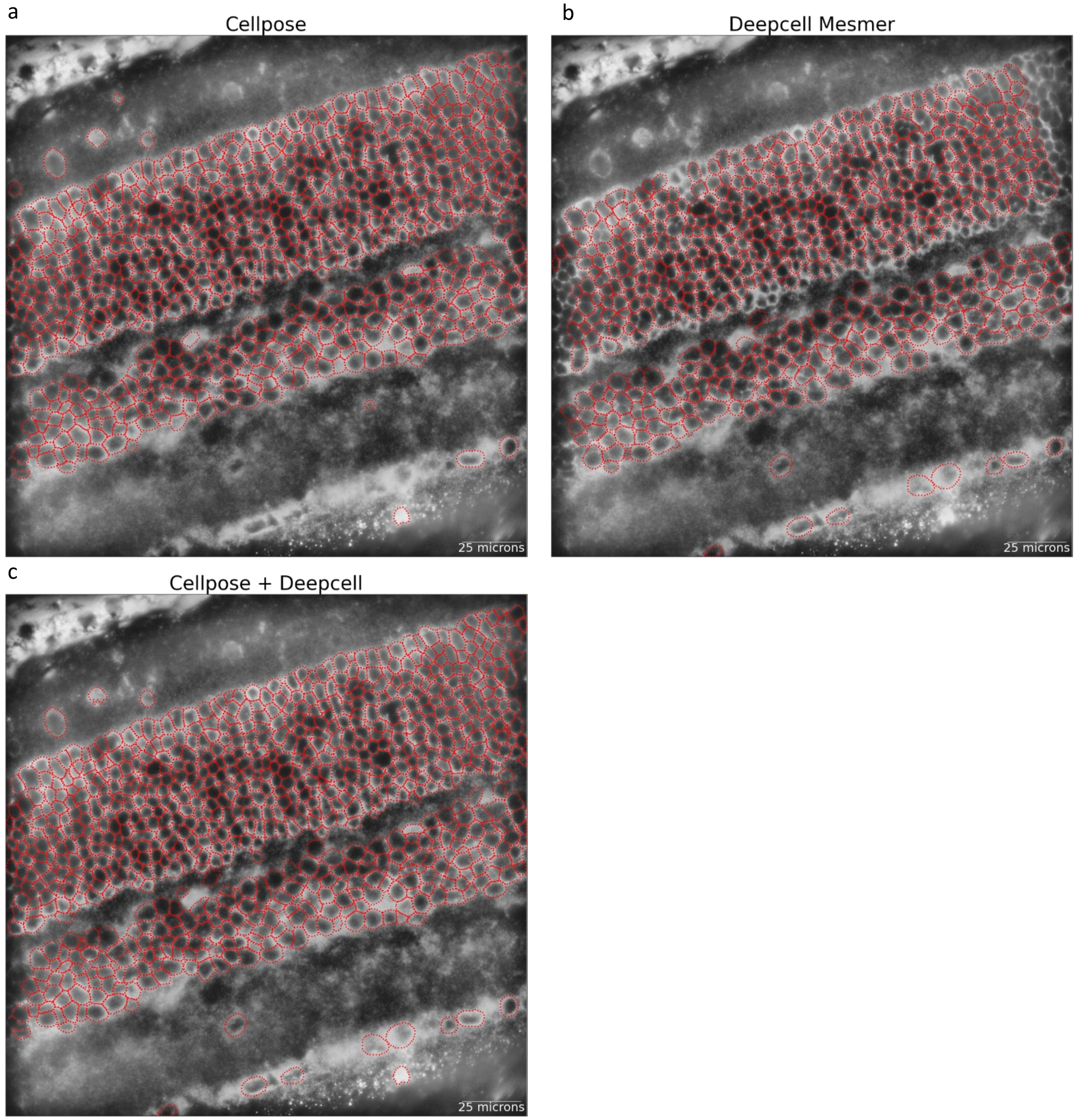

Supplementary Figure 2. Segmentation results in the three retinal layers by Cellpose and Mesmer algorithms. a, Cellpose performs well in the ONL and INL, but fails in the GCL. **b,** Mesmer often under-segments and results in multiplets in the INL but performs well in the GCL. **c,** Combination of Cellpose and Mesmer. Cellpose result was used as the base with most ONL and INL cells properly segmented. Mesmer results were used to fill in areas with missing segmentation, mainly in the GCL. This representative field of view shows similar staining and segmentation results as other fields of view used in the analysis.

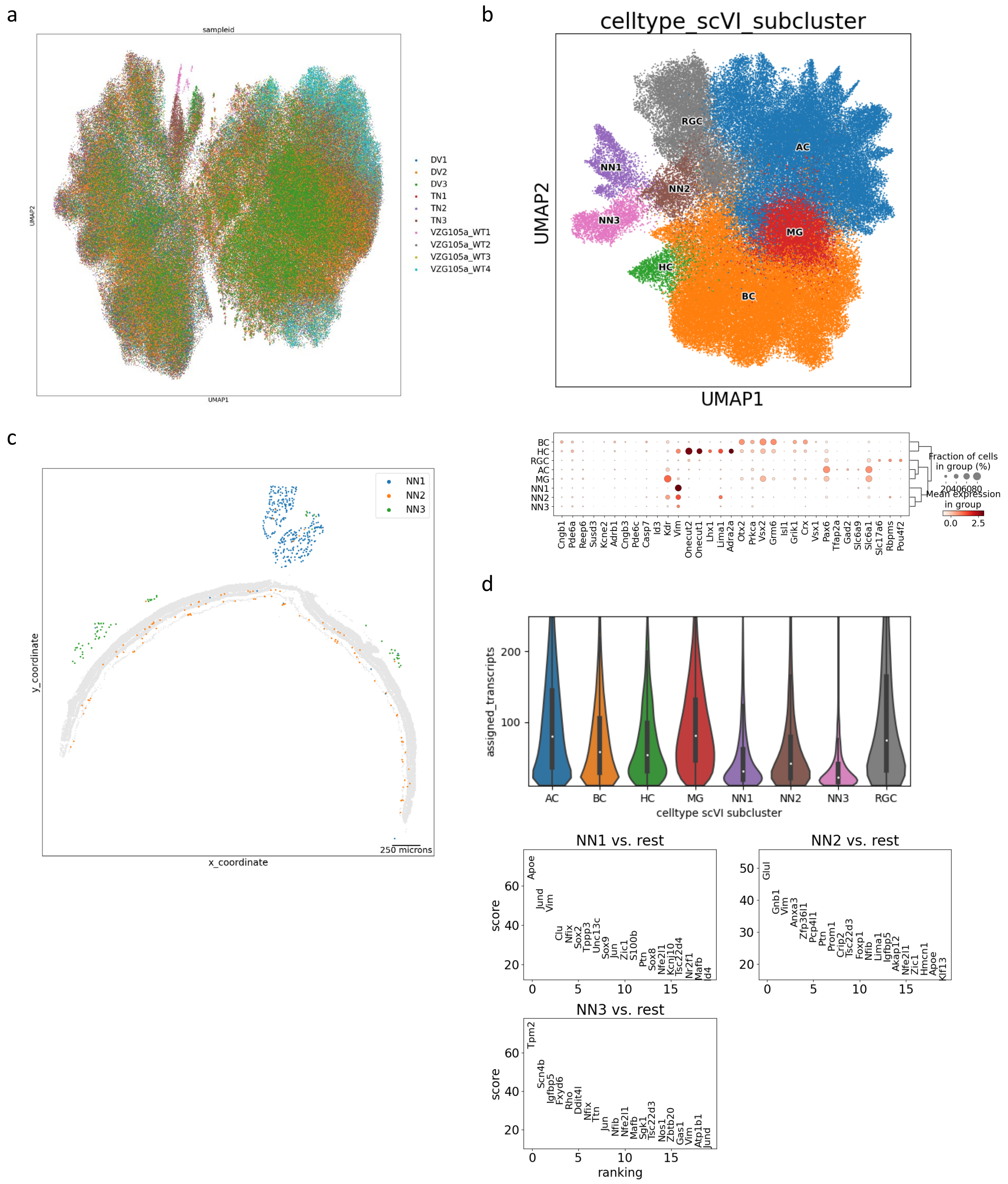

Supplementary Figure 3. Integrative clustering results of 10 MERFISH experiments and cell coordinates of non-neuronal cell type. a, Integrative clustering analysis of MERFISH experiments. Batch correction between ten MERFISH experiments using two different probe panels resulted in even distribution of cells with strong overlap. **b,** Sub-clustering analysis of non-photoreceptor cells. A subset of non-photoreceptor cells was clustered to annotate 5 retinal clusters and 3 non-neuronal clusters. **c,** Distribution of non-neuronal retinal cell types. Three

non-neuronal cell clusters showed localization in the optic nerve, retina, and outside of the RPE/retinal layers. **d,** Transcript count in non-retinal clusters compared with retinal clusters and differential gene expression of non-neuronal cell clusters (n = 10 independent experiments). The bounds of the boxes represent 25 to 75% percentiles with the center lines showing the median. The whiskers extend 1.5 times beyond inter-quartile ranges. Non-neuronal clusters showed significantly decreased transcript counts compared to retinal clusters. The cellular localization and DEGs of each non-neuronal cluster suggest that they are likely oligodendrocyte, microglia/astrocytes, and muscle cells.

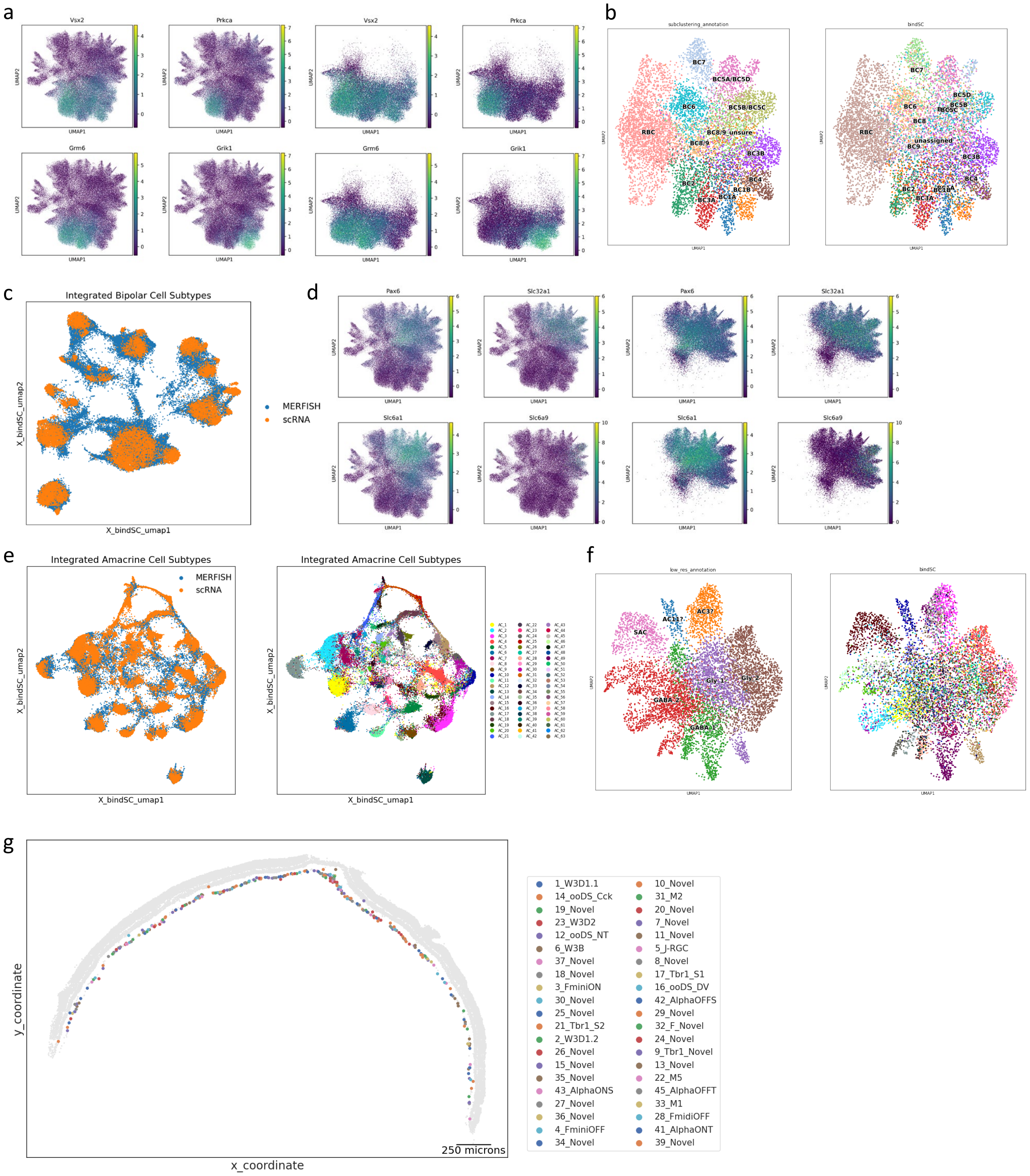

Supplementary Figure 4. ON and OFF types of bipolar cells, bipolar subtype clustering analysis based on MERFISH panel genes, overlap between MERFISH and scRNA-seq bipolar cells, GABAergic and glycinergic types of amacrine cells, overlap between MERFISH and scRNA-seq amacrine cells, amacrine subtype clustering analysis based on MERFISH panel genes, cellular distribution of annotated RGC subtypes. a, Bipolar

subgroup marker expression. A pan-BC marker Vxs2 is expressed across all BCs. BC subgroup markers such as Grm6 for ON cone BC, Grik1 for OFF cone BC, and Prkca for RBC are expressed specifically within the substructures of BC clusters. **b,** BC subtype label overlaid on the lower dimensional space calculated using only 368 MERFISH features. Clustering analysis with only the MERFISH features results in poor resolution, as shown in the clustering map with merged subtypes on the left. Overlaying the subtype annotation from co-embedding analysis on the same map shows separation within previously merged subtypes such as BC5A/BC5D in the right plot. **c,** Co-embedding plot of BC from MERFISH and scRNA-seq. BCs identified in MERFISH (blue) and scRNA-seq (orange) show strong overlap. **d,** Amacrine subgroup marker expression. Pan-AC markers Pax6 and Slc32a1 are broadly expressed across all ACs. Expression of GABA and glycinergic receptor markers, Slc6a1 and Slc6a9 were confined within substructures of AC clusters. **e,** Co-embedding plot of amacrine cells from MERFISH and scRNA-seq. ACs identified in MERFISH (blue) and scRNA-seq (orange) show reasonable overlap. **f,** AC subtype label overlaid on the UMAP with 368 MERFISH features. Clustering analysis using the MERFISH features does not provide sufficient resolution to identify AC subtypes as shown in the left plot. The co-embedding annotation on the same UMAP on the right shows that AC subtypes are confined within substructures of clusters, represented by clumped up cells in the same colors. **g,** Tissue plot of identified retinal ganglion cell subtypes. All RGCs are seen in the GCL, where they are known to reside.

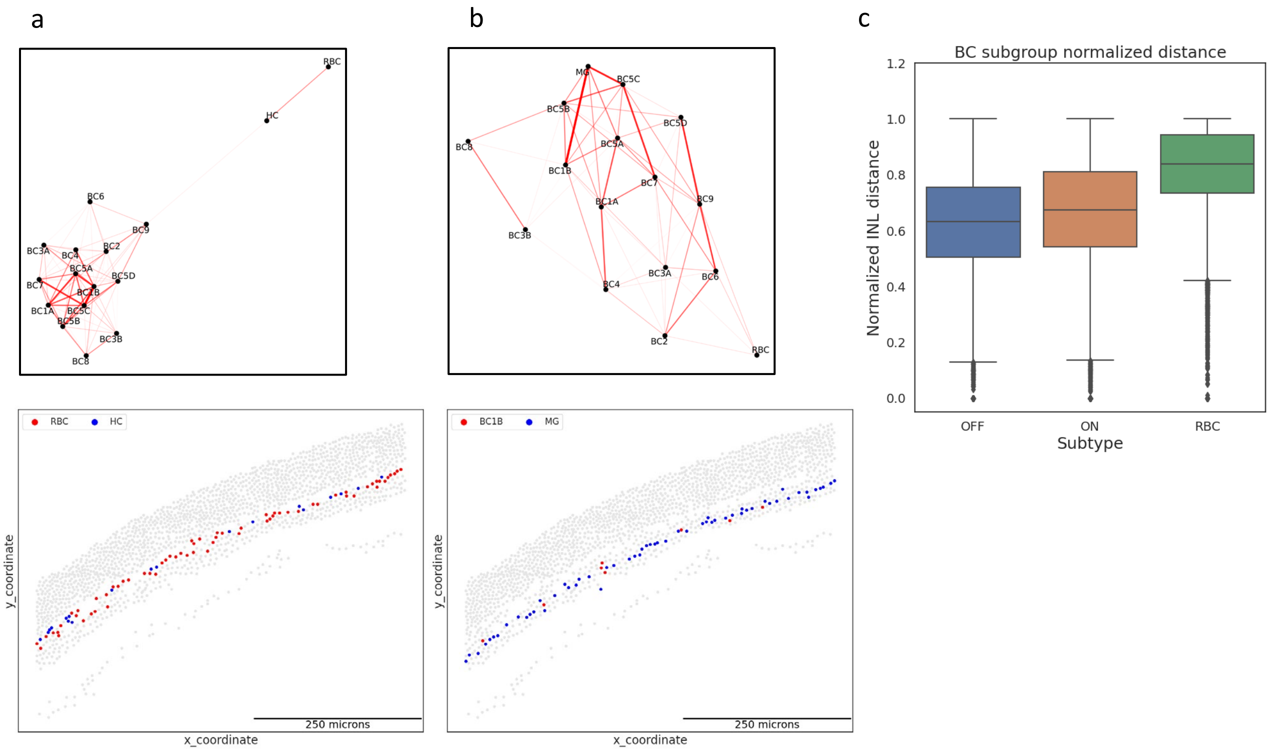

Supplementary Figure 5. Spatial relationship between RGC and HC, spatial relationship between BC1B and MG, average INL position of OFF, ON, and rod bipolar cells. a, Spatial relationship between rod bipolar cells and horizontal cells. RBC and HC show high proximity enrichment scores as shown in the network plot on top. The tissue plot at the bottom indicates both RBC and HC are positioned in the apical layer of the INL. **b,** Spatial relationship between BC1B and Muller Glial cells. Network plot on top show high proximity enrichment score for BC1B and MG. The tissue plot at the bottom indicates BC1B are positioned in the central INL along MG. **c,** Average distance of OFF cone, ON cone, and RBC in the normalized INL (n = 10 independent experiments). No significant difference between OFF and ON cone BC distance was found. RBCs show significant apical positioning compared with cone BCs. In the box plots, the bounds of the boxes represent 25 to 75% percentiles with the center lines showing the median. The whiskers extend 1.5 times beyond inter-quartile ranges. Individual points determined to be outliers are visualized outside of the whiskers.

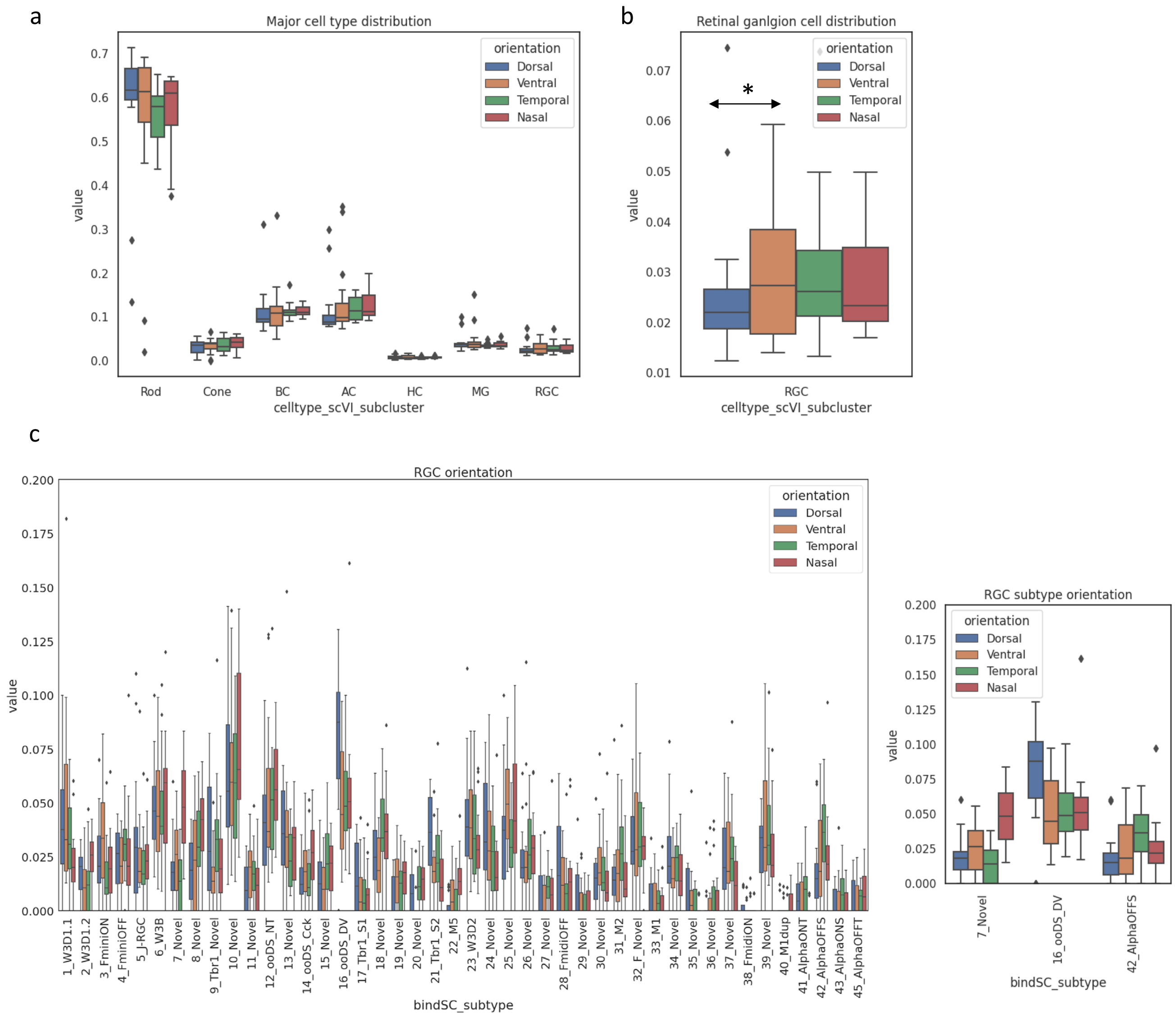

Supplementary Figure 6. Distribution of retinal cells in the four retinal domains. a, Major cell type distribution pattern. No significant distribution patterns were observed across dorsal-ventral and temporal-nasal region, other than a subtle decrease of RGC in the dorsal region. **b,** RGC distribution patterns. A slight decrease of RGCs in dorsal region was observed (p-value < 0.05, two-sided Student's t-test). **c,** RGC subtype distribution patterns. All cell type and subtype population ratios were examined over 16 nasal-temporal sections and 15 dorsal-ventral sections each across 3 independent experiments. In the box plots, the bounds of the boxes represent 25 to 75% percentiles with the center lines showing the median. The whiskers extend 1.5 times beyond inter-quartile ranges. Individual points determined to be outliers are visualized outside of the whiskers.

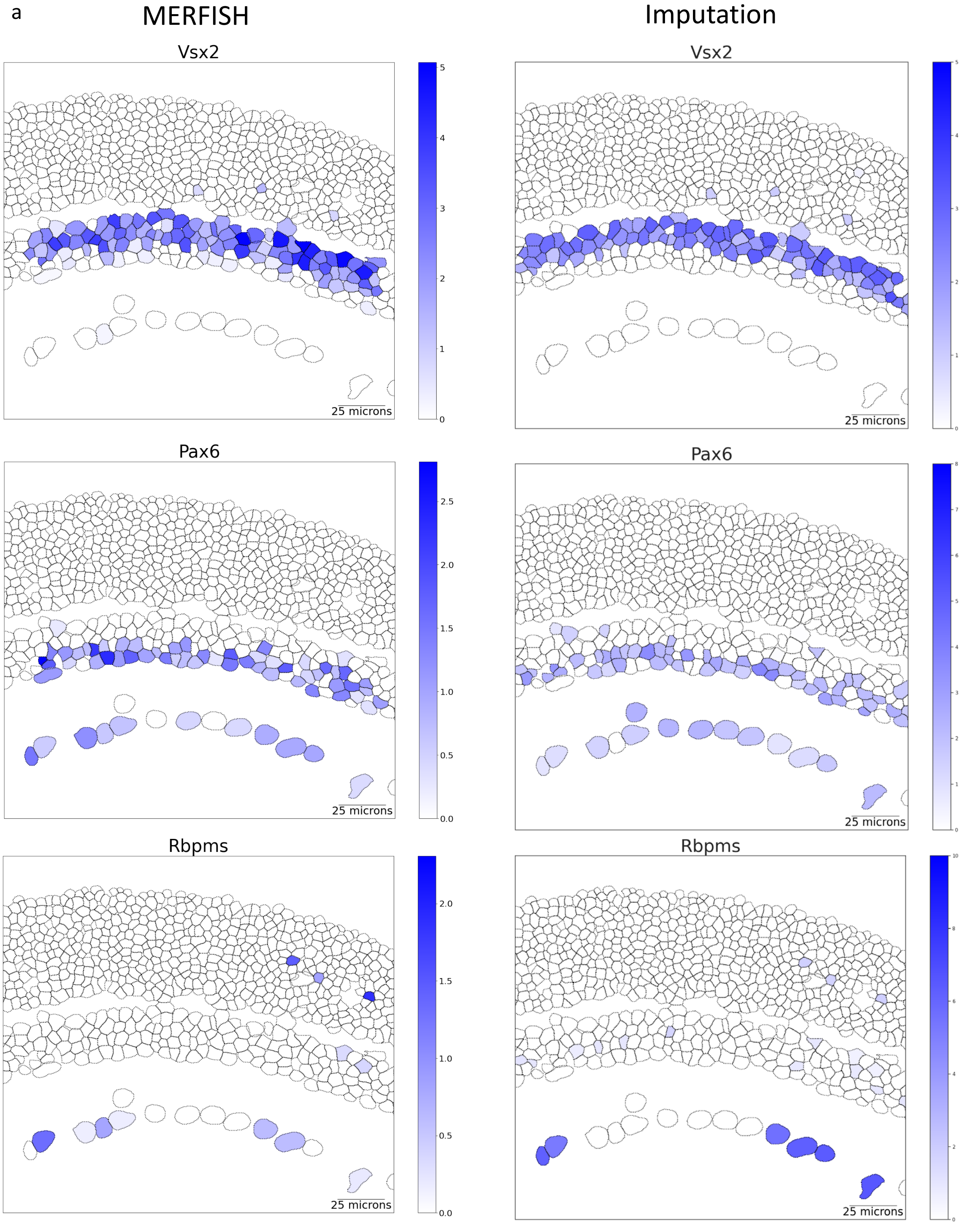

Supplementary Figure 7. Inferred gene expression subtypes. a, Spatial gene expression patterns of cell type markers in MERFISH profiling and imputation. Cell type markers such as

Vsx2 for BC, Pax6 for AC, Rbpms for RGC were profiled in MERFISH experiments, which also show a proper spatial pattern in the imputed transcriptome.

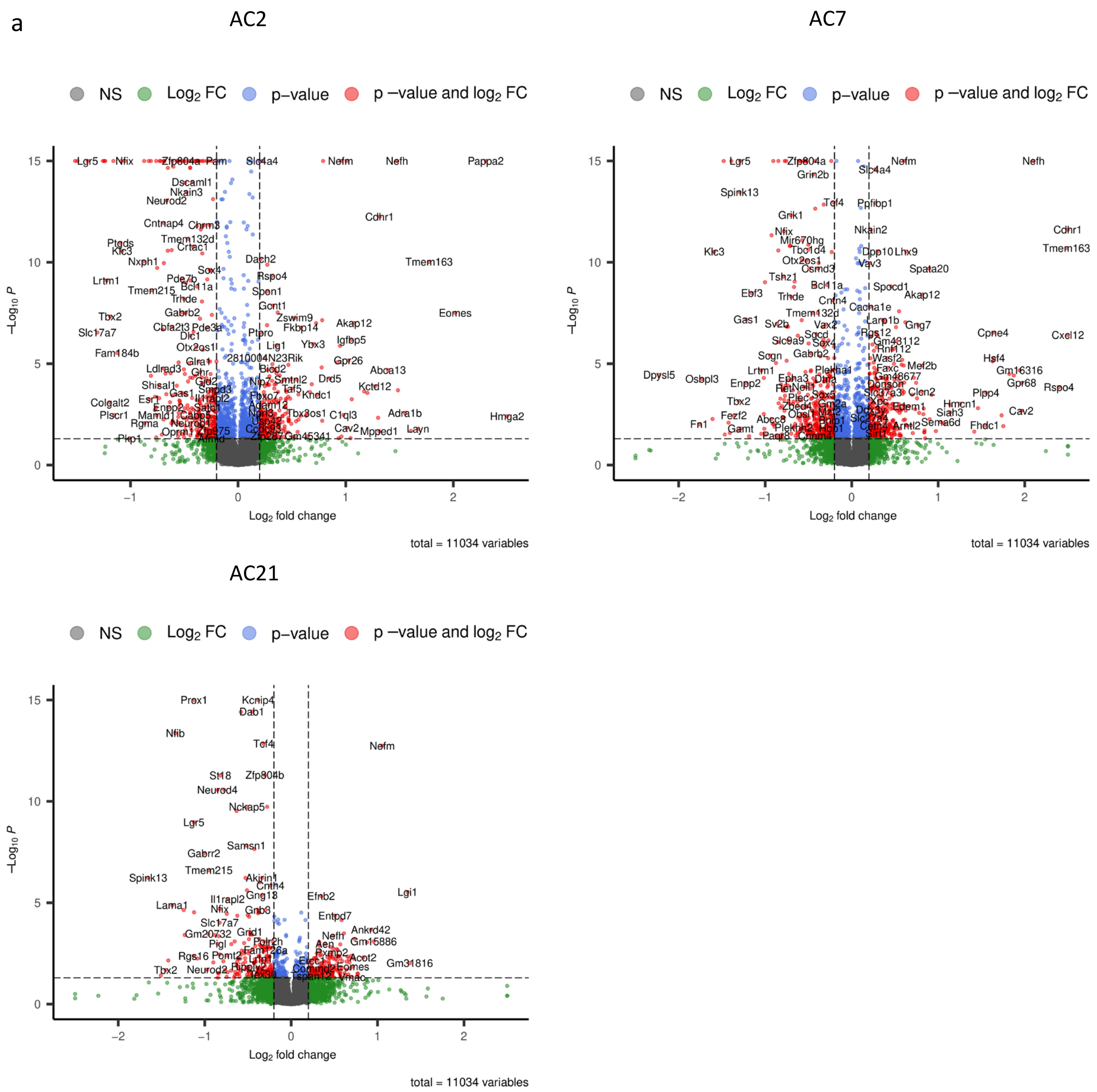

Supplementary Figure 8. DEGs of individual AC subtypes located between INL and GCL. a, Volcano plot of differentially expressed genes in AC2, AC7, and AC21 located between INL and GCL. Differential gene-set enrichment analysis was performed by the DESeq2 package. The p-values are calculated by the likelihood ratio test and adjusted by the Benjamini-Hochberg procedure (see methods).

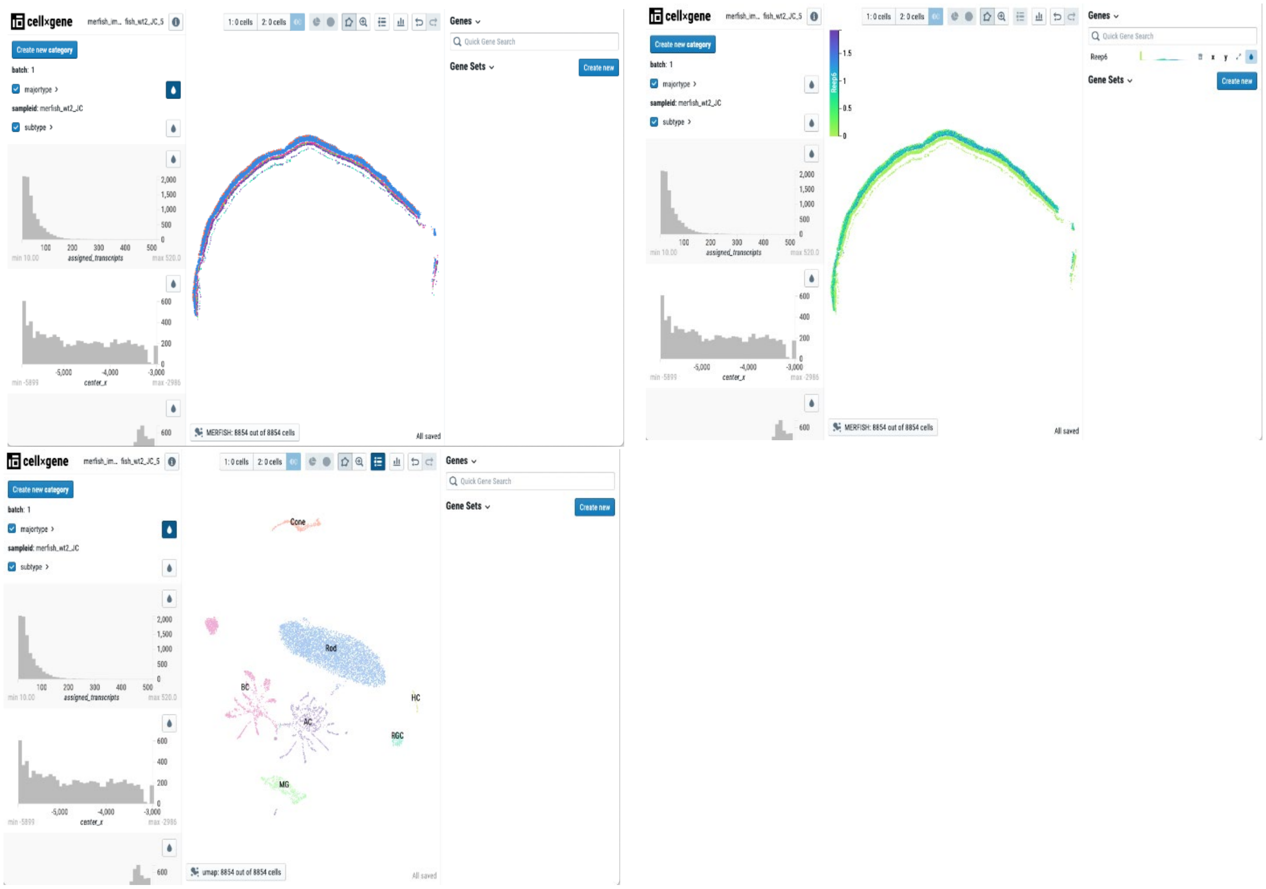

Supplementary Figure 9. Data resource visualization.
