# Supplementary material for: Spatial organization of the mouse retina at single cell resolution by MERFISH
Source: Nat Commun. 2023 Aug 15;14:4929. doi: 10.1038/s41467-023-40674-3 (PMC10427710; doi:10.1038/s41467-023-40674-3)
Supplement: Supplementary file 3 — Description of Additional Supplementary Files [file 41467_2023_40674_MOESM3_ESM.pdf]

Description of Additional Supplementary Files

File Name: Supplementary Data 1
Description: Lists of genes included in the MERFISH probe panels

File Name: Supplementary Data 2
Description: Gene set enrichment analysis result of spatially dependent genes in non-displaced and displaced AC subtypes
