# Supplementary material for: Spatial organization of the mouse retina at single cell resolution by MERFISH
Source: Nat Commun. 2023 Aug 15;14:4929. doi: 10.1038/s41467-023-40674-3 (PMC10427710; doi:10.1038/s41467-023-40674-3)
Supplement: Supplementary file 6 — Reporting Summary [file 41467_2023_40674_MOESM6_ESM.pdf]

nature portfolio

Corresponding author(s): Rui Chen

Last updated by author(s): Jul 13, 2023

Reporting Summary

Nature Portfolio wishes to improve the reproducibility of the work that we publish. This form provides structure for consistency and transparency in reporting. For further information on Nature Portfolio policies, see our Editorial Policies and the Editorial Policy Checklist.

Statistics

For all statistical analyses, confirm that the following items are present in the figure legend, table legend, main text, or Methods section.

n/a	Confirmed	
☐	☒	The exact sample size (*n*) for each experimental group/condition, given as a discrete number and unit of measurement
☐	☒	A statement on whether measurements were taken from distinct samples or whether the same sample was measured repeatedly
☐	☒	The statistical test(s) used AND whether they are one- or two-sided<br>*Only common tests should be described solely by name; describe more complex techniques in the Methods section.*
☐	☒	A description of all covariates tested
☐	☒	A description of any assumptions or corrections, such as tests of normality and adjustment for multiple comparisons
☐	☒	A full description of the statistical parameters including central tendency (e.g. means) or other basic estimates (e.g. regression coefficient) AND variation (e.g. standard deviation) or associated estimates of uncertainty (e.g. confidence intervals)
☐	☒	For null hypothesis testing, the test statistic (e.g. *F*, *t*, *r*) with confidence intervals, effect sizes, degrees of freedom and *P* value noted<br>*Give P values as exact values whenever suitable.*
☒	☐	For Bayesian analysis, information on the choice of priors and Markov chain Monte Carlo settings
☒	☐	For hierarchical and complex designs, identification of the appropriate level for tests and full reporting of outcomes
☐	☒	Estimates of effect sizes (e.g. Cohen's *d*, Pearson's *r*), indicating how they were calculated

Our web collection on statistics for biologists contains articles on many of the points above.

Software and code

Policy information about availability of computer code

Data collection: Custom python codes provided by Vizgen were used to generate the MERFISH images.

Data analysis:
MERFISH analysis:
Merlin 0.1.9, provided by Vizgen, was used to decode the barcoded transcripts from the raw images in MERFISH experiments.
Cellpose 0.6.5 and Deepcell mesmer 0.10.0 were used to segment cell boundary staining images.
Custom python code using SciPy package was used to assign transcripts to segmented cells for generating count matrices.
Scanpy 1.8.1 and scVI 0.6.8 were used to perform single-cell clustering analysis and integration of spatial datasets.
BindSC 1.0.0 was used to perform co-embedding and integration MERFISH and reference scRNA-seq datasets to predict MERFISH subtype classification.
Tangram 1.0.2 was used to impute inferred gene expression.
CellxGene (vl.0.0) was used to generate use-accessible data resources.

Immunofluorescence data:
Quantification was performed by manual counting using Adobe Photoshop.

The code for MERFISH image analysis can be accessed on Github (https://github.com/RCHENLAB/SpatialMmMERFISH) and Zendo (https://doi.org/10.5281/zenodo.8143414).

For manuscripts utilizing custom algorithms or software that are central to the research but not yet described in published literature, software must be made available to editors and reviewers. We strongly encourage code deposition in a community repository (e.g. GitHub). See the Nature Portfolio guidelines for submitting code & software for further information.

Data

Policy information about availability of data

All manuscripts must include a data availability statement. This statement should provide the following information, where applicable:

- Accession codes, unique identifiers, or web links for publicly available datasets
- A description of any restrictions on data availability
- For clinical datasets or third party data, please ensure that the statement adheres to our policy

Mouse retina MERFISH data have been deposited and are available at Zendo (https://doi.org/10.5281/zenodo.8144355). The MERFISH count matrices are in the .h5ad format, which includes metadata such as annotated cell type labels and coordinates of segmented cells. All other data used in this study are available from the corresponding author on requests.

Three previously published scRNA-seq datasets were used as reference: (1) Shekhar, K. et al. Comprehensive Classification of Retinal Bipolar Neurons by Single-Cell Transcriptomics. Cell 166, 1308-1323.e30 (2016), (2) Yan, W. et al. Mouse Retinal Cell Atlas: Molecular Identification of over Sixty Amacrine Cell Types. J Neurosci 40, 5177-5195 (2020), and (3) Tran, N. M. et al. Single-Cell Profiles of Retinal Ganglion Cells Differing in Resilience to Injury Reveal Neuroprotective Genes. Neuron 104, 1039-1055.e12 (2019).

Research involving human participants, their data, or biological material

Policy information about studies with human participants or human data. See also policy information about sex, gender (identity/presentation), and sexual orientation and race, ethnicity and racism.

Reporting on sex and gender	n/a
Reporting on race, ethnicity, or other socially relevant groupings	n/a
Population characteristics	n/a
Recruitment	n/a
Ethics oversight	n/a

Note that full information on the approval of the study protocol must also be provided in the manuscript.

Field-specific reporting

Please select the one below that is the best fit for your research. If you are not sure, read the appropriate sections before making your selection.

☒ Life sciences ☐ Behavioural & social sciences ☐ Ecological, evolutionary & environmental sciences

For a reference copy of the document with all sections, see nature.com/documents/nr-reporting-summary-flat.pdf

Life sciences study design

All studies must disclose on these points even when the disclosure is negative.

Sample size	No sample-size calculation was performed. The sample sizes were chosen based on previous literature with similar experimental paradigms (DOI: 10.1126/science.aau5324; DOI:10.1038/s41586-021-03705-x).
Data exclusions	No data were excluded.
Replication	Four replicates of MERFISH experiments were done without the anatomical orientation information. Three replicates of MERFISH experiment in each dorsal-ventral and temporal-nasal orientation were done.<br>RNAScope experiments were performed twice.<br>Immunofluorescence experiments were performed three times.
Randomization	Eye tissues were randomly allocated for experiments.
Blinding	Blinding was not applicable to MERFISH, RNAScope and immunofluorescence experiments since global profiling of transcripts, unbiased method in cell type annotation and within-sample comparison were performed.

Reporting for specific materials, systems and methods

We require information from authors about some types of materials, experimental systems and methods used in many studies. Here, indicate whether each material, system or method listed is relevant to your study. If you are not sure if a list item applies to your research, read the appropriate section before selecting a response.

Materials & experimental systems

n/a	Involved in the study
☐	☒ Antibodies
☒	☐ Eukaryotic cell lines
☒	☐ Palaeontology and archaeology
☐	☒ Animals and other organisms
☒	☐ Clinical data
☒	☐ Dual use research of concern
☒	☐ Plants

Methods

n/a	Involved in the study
☒	☐ ChIP-seq
☒	☐ Flow cytometry
☒	☐ MRI-based neuroimaging

Antibodies

Antibodies used	Primary antibody: anti-Prkca (Sigma P4334)<br>Secondary antibody: Cy5 Donkey anti-Rabbit IgG (Jackson ImmunoResearch 711-175-152)
Validation	anti-Prkca (Sigma P4334): This antibody has been used to stain for rod bipolar cells in mouse retina in many previous publications such as Inner retinal preservation in the photoinducible I307N rhodopsin mutant mouse, a model of autosomal dominant retinitis pigmentosa. Antonia Stefanov et al. The Journal of comparative neurology, 528(9), 1502-1522 (2019-12-08)<br><br>Secondary antibody was validated by the manufacturer<br>Cy5 anti-Rabbit https://www.jacksonimmuno.com/catalog/products/711-175-152

Animals and other research organisms

Policy information about studies involving animals; ARRIVE guidelines recommended for reporting animal research, and Sex and Gender in Research

Laboratory animals	Mus musculus, C57Bl/6J, 3 months. C57Bl/6J mice were bred in-house and maintained in a 14h light/ 10h dark cyclic environment with the temperature 20±2°C and relative humidity 50±5%. Animals were housed by Baylor College of Medicine Center of Comparative Medicine.
Wild animals	The study did not involve wild animals.
Reporting on sex	Sex-based analysis was not performed as we expect no difference in retinal tissue or cell organization between male and female mice.
Field-collected samples	The study did not involved field-collected samples
Ethics oversight	Mouse housing, experiments, and handling were approved by the Baylor College of Medicine Institutional Animal Care and Use Committee, and the studies were conducted in adherence with the ARVO Statement for the Use of Animals in Ophthalmic and Vision Research and followed the guidance and principles of the Association for Assessment and Accreditation of Laboratory Animal Care.

Note that full information on the approval of the study protocol must also be provided in the manuscript.
